# “I don't know how, if, it's ever going to end”: narratives of caring for someone with an enduring eating disorder

**DOI:** 10.1007/s40519-024-01681-5

**Published:** 2024-07-30

**Authors:** Charlotte Burman, Paul Rhodes, Sabina Vatter, Jane Miskovic-Wheatley

**Affiliations:** 1 School of Psychology, The University of Sydney, Sydney, Australia; 2 InsideOut Institute, University of Sydney and Sydney Local Health District, Sydney, Australia

**Keywords:** Carers, Eating disorders, Severe and enduring, Lived experience, Qualitative research, Narrative inquiry

## Abstract

**Background:**

Families and carers are pivotal in supporting loved ones experiencing eating disorders. This role can bring immense distress and burden, yet the experience of caring for someone with an enduring eating disorder has had minimal research focus. Thus, the purpose of this study is to give voice to carers empowering their stories to increase awareness and understanding, which could inform support to carers and consequently people with a lived and/or living experience of eating disorders.

**Methods:**

Semi-structured interviews were conducted with 9 carers supporting individuals who had been experiencing an eating disorder for 7 or more years. Data were collected and analysed using narrative inquiry approach.

**Results:**

Carers’ narratives revealed feelings of guilt and personal failure; a profound sense of disillusion with current treatment approaches; and immense grief and anguish. As they negotiated a tenuous relationship with hope and the uncertainty of their loved one’s future, carers spoke to a complex myriad of feelings of acceptance, letting go, and forging on.

**Conclusion:**

Carers deserve to have their voices heard where they are too often silenced. Their narratives provide an urgent call for transformation in our treatments for eating disorders and further involvement of carers within the treatment journey, and their lived experience perspectives have great potential to guide this endeavour.

**Level of Evidence:**

Level V, qualitative interviews.

**Supplementary Information:**

The online version contains supplementary material available at 10.1007/s40519-024-01681-5.

## Introduction

Eating disorders (EDs) are serious psychiatric illnesses associated with profound psychosocial impacts and alarming physical risks for the person, and significant societal costs [1–4]. Families, carers and supports play a pivotal role in supporting those living with EDs [5] and are often the primary treatment resource [6–8]. Clinical outcomes have been demonstrated to be improved with carer involvement [9]; however, carer voices are frequently neglected in clinical and research contexts [10]. Many EDs show a protracted course, yet to date, there is a paucity of research examining the lived experience of caring for someone with a long-standing ED [11].

Caring for someone with an ED can bring a high level of distress and burden [12, 13]. For example, family-based treatment (FBT) for child/adolescent anorexia nervosa (AN) requires substantial commitment from carers who are expected to dedicate much of their lives to the intensive refeeding of their child [14–16]. For many carers, this support requires such immense time and energy they often give up work, study, social and leisure activities [17]. The demands of caregiving often become all-consuming and can impact the capacity to attend to other relationships, potentially contributing to family conflict and dysfunction; resentment from those who feel neglected; and social isolation for the carer [17, 18].

To date, only a few studies [15] have examined carer experiences in the context of less successful treatment attempts, considered duration of caregiving, or examined the experience in context of long-standing illness. Approximately 20% of individuals experiencing AN develop persistent and serious illness—severe and enduring anorexia nervosa (SE-AN) (defined by illness duration > 7 years and several unsuccessful treatment attempts [19]). At 22-year follow-up, AN recovery rates have been reported at < 63% [20]. Understanding and supporting the carer’s experience in the long term is vital for the long-term wellbeing of themselves and the person they are caring for.

Involving individuals with lived experience in research is crucial as it provides opportunity to reclaim the narrative, restore a sense of personal justice, and move from marginalisation to feeling valued [21], whereas failing to listen precludes us from understanding, learning from, and supporting lived experience. This study seeks to respond to these important objectives by using a narrative inquiry framework to investigate the experience of caring for someone with a long-standing ED, to offer insight into how caregiving evolves over time and what it is like when treatment is less successful or relapse occurs, when illness or distress endures, and when new illness phases and life stages must be navigated. Importantly, the qualitative approach empowers carers to define their own stories and emphasises the meanings they ascribe to their experiences [22] and these narratives may offer important insights to consider while shaping future research and therapy paradigms.

## Method

### Participants

Participants were recruited from a research project evaluating an online skills-based programme for carers of people with EDs and had previously consented to being contacted [23]. Purposive sampling identified individuals who self-identified as the primary carer for someone with ED symptomatology for 7 or more years. Type of carer, age of the person they were caring for or ED diagnosis was not limited. In total, 21 carers were invited to interview, of whom 13 responded and were booked for interviews. One carer did not continue due to work stress, and three others completed interviews; however, their transcripts were excluded from the current analysis as two were carers for significantly less than 7 years, and one was not a primary carer and described limited involvement.

### Design

Data were collected through interviews within a narrative inquiry framework [24] that examines processes occurring over time [25–27]. In creating space for a person to describe in detail the wider context of an experience, narrative inquiry allows for a holistic depiction of phenomena rather than a fragmented account and captures the unfolding dimension of experiences [25, 28]. Through enabling storytelling, narrative inquiry allows people to bring matters of importance to the surface [28]. This approach considers the expertise of lived experience and prioritises participant voice and interpretation in the construction of narratives and has proven to be invaluable within SE-AN research [25, 29].

### Data collection and procedure

Participants were interviewed via videoconferencing (*n* = 7) or by telephone (*n* = 2). A minimalistic interview guide was followed (see Supplementary Material 1), so participants could freely develop their own account and capture the complex and varied elements of each story allowing them to have control of event descriptors and links, while the interviewer needs to give up control [28]. Probing and prompting questions were used to elicit further details to produce a descriptive timeline of experience from the start of their caregiving towards future thinking.

Audio-recorded interviews ranged from 45 min to 2 h and were transcribed verbatim. Narrative summaries were created such that data from the transcripts were placed in temporal order to create a chronological story [28]. Throughout narrative construction, direct quotes were used to preserve carers’ voices, with minimal additions made to support the flow of the narrative.

### Analysis

Analysis was guided by the narrative inquiry procedure outlined by Howie [28]. Each narrative was first analysed on a line-by-line basis. Main themes were identified to capture the central issues relevant to each story and narratives were synthesised to a timeline outlining the caregiving journey with themes being positioned in temporal context. This allowed commonalities across narratives to be examined and for a collective story to be composed. Throughout data collection, coding and analysis, memo writing was used to capture investigator’s reflections.

Member checking and collaborative coding were used to enhance rigour and trustworthiness of the data [24], and investigators met throughout analysis to confirm the attribution of themes to stories and create thematic structure; a thorough audit trail was kept ensuring analytic transparency. Member checking involved emailing participants a draft analysis for review, inviting feedback and/or summary amendment, to ensure the narrative appropriately reflected their experiences. Five carers responded, all confirming that the narratives were an accurate representation of their experiences; one carer indicated a possible addition to the results, which was included.

## Results

### The sample

Nine carers took part of whom 8 were mothers and one was a partner (see Table 1). Carers were supporting individuals of varying ages who had been experiencing either AN (*n* = 8) or avoidant restrictive food intake disorder (ARFID, *n* = 1) for 7–15 years. To note, the latter case was included as upon review their overall experience (including treatment) was very similar to the experiences of the rest of the cohort.

**Table 1:** Characteristics of carers and the individuals they cared for

Carer details		Characteristics of person with ED		
Pseudonym	Relationship to person with ED	Years experiencing ED symptoms	Age range	Type of ED
Teresa	Mother	7	15–19	AN
Lily	Mother	8	20–24	AN
Leah	Mother	8	15–19	AN
Daniel	Partner	10	45–49	AN
Julia	Mother	10	15–19	ARFID
Kate	Mother	10	15–19	AN
Sophia	Mother	13	30–34	AN
Maria	Mother	14	25–29	AN
Esther	Mother	15	25–29	AN

*AN* Anorexia Nervosa; *ARFID* Avoidant Restrictive Food Intake Disorder; *ED* Eating Disorder

### Findings

Carers began their stories by recounting events that led up to the illness onset including ways they contributed to, or failed to prevent, the development of their loved one’s ED describing early warning signs missed and regret about not intervening sooner. From the beginning, a sense of carer guilt emerged.

#### “We had no clue what had hit us” (Leah)

Carers recalled their initial reactions as reflecting an innocent naivety about what lay ahead. Many reported laissez-faire attitudes attributing to their limited understanding of EDs. Daniel *“didn’t think much of it”* and Kate *“assumed things would be right”.* Some felt that the severity of the condition was not appropriately conveyed to them at this time; *“Had we understood that it was the most lethal of mental illnesses, our whole demeanour would have been different, but we weren't told that.”* (Kate).

Others reported a building sense of panic as their intuitions about the problem’s severity were met with ambivalence from doctors who dismissed their concerns. Teresa, Lily and Sophia spoke of going through a *“string of GPs [General Practitioners]”* (Sophia) who demonstrated very limited knowledge of EDs, obstructing timely diagnosis. Many felt that the realities of their loved one’s illness were not comprehended by health professionals, yet carers’ attempts to convey their concerns were dismissed as exaggeration or falsehood.

*“I can distinctly remember one doctor talking to me like an infant and saying, “Mrs, you need to understand that no child will willingly starve themselves. I think you need to go and get the treatment.” And that left me like a shag on a rock.” (Sophia)*

#### “What do we do? Where do we go?” (Lily)

For many, diagnosis did not come with adequate education, support, or guidance. When Teresa’s daughter was first diagnosed, she was simply instructed to *“take her home and feed her”*. Though this task seemed simple, carers faced resistance at every stage. Lily became increasingly desperate as she was unable to access treatment due to paucity of ED-informed professionals in her area: “*I knew there was a problem, but we just couldn't get the help.”*

Many carers blamed themselves for their behaviour when uninformed; Julia felt she *“should have been better”* and Maria described she would do *“so many things differently”* in hindsight. This self-blame amplified as their support efforts appeared fruitless. Daniel felt a *“pressure to be the solution”* as he could not secure professional support for his wife despite her continued decline.

### “The only time I felt any real sense of relief was when she went to hospital.” (Daniel)

When treatment was finally accessed, a sense of relief set in. Hospital admissions signalled to carers that their concerns were being taken seriously and offered respite from the intensity of caregiving. Esther described her daughter’s first admission as *“such a relief”* because *“somebody else [was] looking after her*”. Daniel identified his wife’s admission as a time of optimism and hope for recovery: “*she’s going to get treatment (…) and hopefully, she’s going to come out better”.*

### “She absolutely came out better” (Daniel)

Some carers reported positive experiences of treatment. Lily pinpointed an inpatient admission as *“possibly the turning point”* in her daughter’s journey. Daniel noted that although it was the fear of returning to hospital that drove eating restoration in his wife, he nonetheless “*felt really true hope that she could recover.”* For Esther, her daughter’s progress was so marked that when reminded of the often-protracted course of ED treatment, she thought *“Oh, this must be the exception”* and counted herself amongst the lucky few.

### “So, it's not as easy as we thought…” (Esther)

After her initial gains, Esther’s daughter began to decline and needed to be readmitted to hospital, a pattern which began to recur. Daniel was *“devastated”* and plagued with guilt when he witnessed his wife’s hard-won progress had again been lost*.*

For those carers supporting an adolescent with AN, intervention took the form of FBT. Though it was presented to them as the ‘gold-standard’ treatment, several attempts at FBT were unsuccessful in achieving weight restoration.

*“She had 30 admissions between when she was 11 and when she was 16 or 17. And they were horrendous. She just never, ever once got past phase one of [FBT]. So, she’s never to this day been weight restored.” (Leah)*

Soon, FBT became unfeasible to sustain, particularly due to impacts on other family members. Carers were frustrated that FBT’s emphasis on weight restoration meant that their adolescent’s psychological distress was not addressed. For Kate’s daughter, inpatient admissions brought opportunities for psychological intervention, however, they were “*missed every time” as* hospitals merely functioned as *“refeeding facilities”.* Teresa’s daughter spent over 2 years in hospital, sleeping on the floor, neglecting her self-care, and refusing to speak with anyone and the only intervention she received was *“palliative care in the form of two nasogastric feeds a day”.*

### “It became a revolving door” (Esther)

Many narratives featured the cyclical pattern of hospital admissions spanning several years, all the while requiring immense sacrifices as carers.

*“When your child is first sick, you remember down to the day how long they’ve been in. But it’s been roughly about 4.5 years’ worth of admissions now. And I’ve been there by her bedside”. (Kate)*

When retelling the beginnings of their stories, carers could pinpoint key moments of devastation and revelation occurring as part of daily caregiving. In discussing this middle phase, several years were condensed and abridged, blurring into tiresome and repetitious cycles giving way to exhaustion, frustration, and disillusion.

*“I see the absolute impotence of current treatment regimes for anorexia. In a few years’ time when there’s more research done on the illness, they’re going to sit back and say, well, effectively, all we did was force-feed them. And that’s hideous.” (Kate)*

### “What are we doing that’s so, so wrong?” (Leah)

As unsuccessful treatment attempts accrued, carers came to blame themselves for the lack of recovery. Leah questioned *“what am I doing wrong?”* as she saw other families “*able to do it in one admission”,* yet her daughter was constantly returning to hospital. Maria spoke to the dissonance between the onus placed on carers and the lack of support provided to them.

*“They believe that parents are the best individuals to refeed their children. That's a huge responsibility and [if] you fail at that, which I feel I did, give me some support. What the hell am I doing wrong? What am I doing right?”*

Teresa felt that the FBT clinician her family worked with *“had no idea what [they] were going through, [only] a textbook she was quoting”.* She reported feeling increasingly frustrated with professionals who pursued the same approaches, which had failed several times, implying a disregard for her lived experience.

Sophia highlighted that the clinical experience of medical professionals cannot substitute carer expertise: *“How would you ever understand something like this? Unless you live with it?”.* Maria similarly implored that carer expertise be utilised more wisely by treating teams: *“They need to listen to the parents. We live with this illness seven days a week.”* Yet there was a sense that carers’ efforts to convey the reality of their situations were frequently disregarded.

#### “You are responsible for everything and not taken seriously by any of the services.” (Teresa)

“It was horrible. The feeling of powerlessness, helplessness. No one listens to you. (…) As a mother you just bear all the responsibility and have no rights. No rights and no say and no respect. And in eating disorders this is so evident because you are asked to do something that no doctor and no nurse can do. If you’re so f*cking smart, come to my home and feed her.” (Teresa)

Though they were relied upon so heavily to spearhead recovery, carers were excluded from decision-making about their loved one’s care and transition to adulthood became a particularly trying time. Julia felt hurt that she was no longer allowed or wanted in appointments*.* Esther similarly came to feel *“on the outside, rather than within the [team]”*. Daniel was uncertain whether to attend his wife’s appointments: *“Do I force myself? Do I just go?”*. Leah highlighted the stark contrast between the child system that *“holds them so close”* and the expectation that when they turn 18 *“they’re magically supposed to be able to do this themselves”.*

#### “You get to rock bottom and where do you go? You just don't know where to turn.” (Lily)

As the inefficacy of dominant treatment approaches became increasingly salient, Leah questioned this path’s logic for her daughter.

*“This is clearly going nowhere. She's done this 30 times. Isn't that the definition of insanity? Doing the same thing over and over again and expecting a different result?”*

Diminished hope in these approaches was coupled with uncertainty about other possible treatments. Sophie recalled the powerlessness of seeing her daughter struggling, yet not knowing what to do or where to seek help. Due to a lack of professional support, carers turned to self-education and advocacy. They “*read nearly every book under the sun” (Lily),* attended *“different conferences” (Teresa),* drove *“doctors mad because [they were] always researching” (Kate)* alternate treatments. Lily wished she could *“just be the carer and not the caseworker”.* As her daughter’s time in hospital stretched on, Teresa began advocating for nasogastric feeding to be done at home and was *“knocking on all the doors to make it happen”,* including writing to the Department of Health, the hospital, and her local member of parliament.

#### “The carer’s journey is incredibly lonely.” (Leah)

Carers reported that friends and family were prone to making unhelpful and misinformed comments leading carers to *“pull away and [become] a bit resentful” (Julia).* For many carers, support from family and friends reached saturation as the years progressed.

*“The people that I’ve relied on have become fatigued (…) (and) their level of support has waned, because it's just all too hard.” (Kate)*

Maria likened the experience to the grieving process, where initially people pay attention and offer their support; however, when she came to desire it, it was no longer on offer.

*“People's lives, they've moved on. People don't want to hear because they've done their thing, in the first two, three months. So, I'm not going to impose, I'm not going to burden anybody with this. It’s my issue, obviously, and I need to deal with it.”*

Some carers reported positive experiences of accessing their own support. Several carers sought professional therapy; others turned to informal self-care. Some found comradery and solidarity within carer support groups. For Lily, other carers became friends because they were “*on the same page”.* Yet many struggled to find belonging in these groups and hesitated to participate in these spaces: “W*hen I tell people I've been doing it for 15 years, it scares everybody.” (Esther).* Daniel found other carers’ stories of recovery “*demoralising”* as they facilitated unhelpful comparisons which compounded his sense of failure.

*“All the negative stuff was really bad, but all the positive stuff was really bad as well, because I didn't feel like it was working for me.”*

#### “Where did we go wrong? Sometimes [we] ask ourselves, why is she still so sick?” (Leah)

The longevity of their loved one’s illness came to be interpreted by carers as evidence of their own culpability, giving way to immense guilt.

*“I know that along the way, there are things that I have done or failed to do to promote recovery. And that's why she has had anorexia for 15 years. I live with that guilt every day. And as much as people can say, well you did the best you could with the resources you had. But it wasn't enough. She's still suffering. I should have been able to do more.” (Maria)*

### “We're just at a loss for what to do.” (Julia)

Waning faith in both themselves as carers and the available treatments options led to despair and uncertainty. Many signalled that caring for an adult brought unique challenges as public health supports became contingent on medical crisis. Sophia, who had survived cancer though feared its recurrence, questioned “*What happens when I'm not here to care anymore?”.* She spoke of the lack of services to meet her adult daughter’s complex needs:

*“There is no support for someone with a severe and enduring eating disorder. Other than when she's on death's door, they’ll accept her into emergency, but there's nobody to support her recovery out of it.”*

Many felt hopeless. Daniel spoke of no longer resonating with messages of hope espoused by other carers: *“I can't buy into that, in the situation I’m in*”. For Kate, a sense of resignation settled.

*“If she's in the 20% category that is treatment resistant, I don't believe a treatment exists on the planet that can help her. We both feel totally deflated.”*

### “I'm grieving what will never be. What they've lost. And who I may lose at some point in time.” (Maria)

Themes of grief permeated stories of caregiving; grief about the years lost and experiences missed out upon, for both the affected individual and the close people. Kate articulated that because *“life has been on hold”* for the 10 years her daughter has been sick, her other daughter lacks “*memories of all the fun things that you should do as a kid with your family.”* Julia spoke of the bonding that occurs over a shared meal and expressed that she *“missed out on all of that”* with her daughter. An easy-going connection with her daughter was difficult for Kate as she was *“her carer but also her jailor”.* However, others did feel that caring had fostered a unique level of closeness in the relationship with their loved one.

Alongside the relational losses, carers described grieving the lives they had envisioned for their loved one. Maria, who cared for two daughters with AN, spoke to the heartache of witnessing their suffering and her fear about their futures.

*“My biggest fear in life for my two girls is that they will die from this illness. And I don't know how to prepare for that. How do you prepare, do anticipatory grieving? I don't even know how to do that.”*

### “I have to learn to let go” (Julia)

Themes of letting go permeated stories of caregiving. Carers conveyed the need to respect the autonomy of their loved one in allowing them to take responsibility for their own recovery.

*“It’s not my recovery. It’s not my journey. It is for her to change this, not for us.” (Maria)*

For Teresa, letting go meant releasing herself from the expectation of needing to save her daughter.

*“I told myself that if she wants to die, as a mother, I have done everything (...) I just have to let her go. I'm not going to live with this forever. I am a loving caring mother. But there is only so much [you can do]. You can't save people, you can only love them.”*

Letting go, for Teresa, was a freeing proposition and entailed reclaiming and celebrating her own life. For Julia, this realisation was fraught with apprehension about what letting go would mean for her daughter.

*“I’ve got to start thinking about me! And that's hard too […] I've got to do this, I’ve got these goals, but what do I do with [her]? Do I just leave her? I can’t just leave her!”*

Many were careful to clarify that letting go did not mean abandoning caregiving, but rather paved the way going forward, one which upheld their own life’s value. Letting go did not always represent an absolute shift, but instead a rebalancing. In envisioning the future caregiving, participants described this process as dynamic and ever evolving: *“It’s like a dance. Sometimes you stay back, and sometimes you move in.” (Esther), “I just have to step back a bit now and let her come forward” (Lily), “She becomes more and I become less” (Leah).* Carers aim to strike a delicate balance between supporting whilst not intruding or rushing in to take charge.

For some, letting go brought fear and uncertainty. Esther felt *“petrified”* and questioned *“how is she going to survive this?”* when her daughter first spoke of moving out of home*.* In anticipating being away for a few months from her daughter, Julia felt a mix of cautious optimism and trepidation: *“Once I’m out of the picture for a while it might change, or it could get a lot worse.”*

#### “Some people say it's scary. I find it quite liberating, actually.” (Leah)

The thought of letting go was a freeing one for many, as they anticipated no longer being bound by such immense caregiving duties. For Lily, a sense of impending relief, however, was muddied with guilt.

*“This sounds really terrible. But after a long battle, it's like, out of sight, out of mind now. Is that terrible? I don't know. I've done this as long as I can do it.”*

For Teresa, radical acceptance offered an alternative to the helplessness she felt.

*“I know it's not over. I know what I can do and what I can't. I don't feel it anymore, that powerlessness.”*

Acceptance implied acknowledging the protracted course of the illness, its likely continuation into the future, and how it had come to represent a new normal. Maria spoke to the ebb and flow nature of the disorder as bringing some sense of precariousness.

*“It's like any chronic illness; you have exacerbation, and you have remissions, and you have stability, instability. […] I'm always waiting for the shoe to fall.”*

Alongside acceptance, some described an inclination to transform their suffering: *“We’ve got to do something with this. We can turn it into a positive.” (Julia)* Though this varied between carers, it often took on an altruistic quality: developing educational resources for other carers; advocating for systems change; envisioning a future where their loved one could be a lived experience coach. Some spoke of adopting a more compassionate worldview, of identifying with belief systems that offered a way to make meaning of their suffering and sustained them through hardship. Sophia expressed a sense of gratitude: *“I'm glad I'm her carer”.* Teresa spoke to how her experiences facilitated personal growth and self-development.

*“It’s the best mentor of my life, anorexia. It's changed my life for the better… It made me wiser. It made me stronger. It made me more knowledgeable. And [now] I am learning how to disrupt systems.”*

Not all carers transformed their suffering and instead they persisted through it with an unwavering resolve. Kate spoke of a determination born from a sense of duty to her daughter: *“We have no choice. It's just one day at a time. And one foot in front of the other.”* Hence, she envisioned pushing onwards: *“I will continue to leave no stone unturned.”* Esther similarly referred to a quiet determination in refusing to give up: *“You just have to keep on trying. You have to press all the buttons. Maybe one of them will turn the light on.”*

For Leah, persistence was maintained by an unwavering hope in recovery.

*“I firmly believe that she will one day be well, and that she will one day live a full life, and I've never wavered on that. So even in her sickest, darkest times I’ve thought no, the best is yet to come for her. And I still firmly believe that.”*

Julia described a more tentative relationship with hope.

*“You've always got this little bit of hope. But it's not really happening. It's really, really hard. Now I just feel sad. Really sad. But I won't give up on her.”*

For Kate, this steadfastness existed alongside a sense of resignation about her daughter’s future;

*“We'll just be keeping [her] alive for as long as we can, because we don't think she's going to make it.”*

These extracts highlight that the journey of caregiving was not straightforward, linear, or uniform. Carers’ narratives demonstrated a holding of tensions: hope together with uncertainty; determination amidst despondency; gratitude alongside despair. In the context of enduring illness, caregiving was as much about letting go as it was forging on, as carers made meaning of what they had endured and negotiated the uncertainty of what lay ahead.

*“I don't know how, if, it's ever going to end for them. Is it possible to recover after 15 years? I believe so. So I hope for them.” (Maria)*

## Discussion

This study centred lived experience perspectives and empowered carers of people with a lived experience of eating disorders to tell their stories. Their narratives emphasise that more needs to be done for this group and offer new insights to shape the transformation of our treatment paradigms to better address the needs of those with enduring EDs, their families, carers and supports.

Carers struggled to negotiate guilt regarding their loved one’s illness and expressed self-contempt for their perceived missteps, though these occurred in a context of limited access to appropriate information and treatments, especially at the time of diagnosis. Previous research supports how ill-informed responses from health professionals compounded carer guilt and contributed to them feeling misunderstood, scrutinised and blamed [18]. Similarly, having carers responsible for refeeding in FBT induces further guilt when weight restoration is not achieved or maintained [15]. Feelings of blameworthiness intensified as evidence-based treatments were unsuccessful and these cycles of attempts and failures risk producing psychological harm and avoidance of future treatments [30].

Dissatisfaction, frustration, and disillusionment with treatment was present across all carer’s narratives, underscoring that approaches that relegate treatment of psychological processes to the latter stages of therapy are failing those who never achieve or maintain medically defined recovery. This is congruent with research demonstrating that patients with EDs resist the reliance on bio-medical indicators of wellbeing and the interpretation of their recovery within a medical discourse [31–33]. Carers described a non-sensical revolving door of futile treatment attempts facilitated by a disregard for unique needs and preferences for more holistic interventions. Indeed, the limited scope of tailored treatments have been identified as a key contributor to treatment discontinuation [33]. The imperative to adapt to both patient and carer values and preferences is an often overlooked element of evidence-based practice in eating disorders [34]. Research evidence, clinical expertise, and patient perspectives together form the ‘three-legged stool’ representing the essential components of evidence-based practice, and effective treatment may be compromised if one of these stool legs is neglected [34].

These findings provide a compelling call for a change in treatment models to better address protracted illness and approaches guided by a *recovery model* may be promising. A recovery model assumes a holistic, person-centred approach that privileges personally meaningful goals identified by the clients emphasising hope, resilience and rehabilitation, rather than symptom resolution [35, 36]. This model has yet to be thoroughly investigated in EDs, though preliminary research seems promising particularly for those who have not responded to symptom-oriented treatments [37, 38].

Carers in this study felt alone and defeated due to being neglected by an inflexible healthcare system that failed to respond to the unique circumstances of their loved one’s illness. High levels of loneliness and isolation among carers have been well documented [13, 18] and previous research has suggested the benefits of belongingness to carer support groups [18]; however, the participants in this study worried about discouraging others from sharing their stories; struggled with unfavourable comparisons; and came to feel alienated by messages of hope and optimism. This indicates a need for support groups stratified by illness stage or duration to increase carers’ sense of solidarity and connection with others who share similar experiences.

This study focused on listening to carers’ voices which are often silenced, especially for those caring for people with long-standing illness. Grief emerged as central to the experience of caregiving and together with a profound sense of disillusionment with treatment options, often leading to resignation and despair. Metaphors of personal transformation and advocacy emerged as strong themes representing how carers gave meaning to their suffering. For others, suffering was not transformed but persisted through an unwavering commitment to their loved one. Much like the stories of women experiencing enduring AN [22], these carers’ narratives were not simple and linear but infinitely nuanced as they refused to betray the complexity of a years-long, multifaceted experience.

### Clinical implications

We urge researchers and clinicians to consider how they might better respond to this complexity. These findings invite us to consider therapeutic interventions that may provide an opportunity for carers to reauthor their stories as ones of love and dedication, rather than of failure and powerlessness and to better address individual preferences and questions of identity negotiation within treatment paradigms [39]; this should extend to both patients and their carers.

Our current interventions, usually operating within a medical model, are experienced as blaming, disempowering, and contributing to psychological harm in this group. Carer distress has a recursive effect on the distress of their loved one (i.e. the distress experienced by a carer affects the distress of their loved one, which in turn affects the carer’s distress again, creating a cycle of mutual influence), though it is also valid and important on its own. If we are to move towards recovery models [10] that better resonate with lived experience perspectives, we must first make space to hear these stories. The narratives of the current study are laden with new insights which are invaluable if we are indeed seeking to *“generate new avenues of exploration*” ([40], p.2) in the field of enduring EDs. Some specific suggestions included valuing carer lived experience at every stage of the healthcare system, support groups customised to the recovery stage, actively involving carers in decision-making, increasing the availability of a variety of treatment options, and moving towards recovery-oriented models of care.

As clinicians, we must consider when it might be judicious to turn towards enhancing quality of life rather than seeking a ‘cure’, whilst maintaining hope and remembering that complete recovery is rare though possible [40]. While facing these important questions, these narratives in this study may guide us.

## Strengths and limits

This study offered valuable new insights with significant clinical implications, though it was not without limitations. Carers’ stories are real to the moment of the interview, not fixed or final as their story continues; however, the trustworthiness of the data was enhanced through the member-checking process. Data analysis was also influenced by the author’s interpretation as a central tenant of qualitative research, however, two authors were senior clinicians with a long history of working with family therapy for AN and were involved in data coding and interpretation to support the authorship team. This exploratory study focused on giving voice to carers, allowing for variation in type, severity and duration of eating disorder, as well as carer relationship, although most carers were parents of a female child with AN and research with diversity in type of carer and EDs is greatly needed. Moreover, the carers were a sub-set interested in completing an online skills-training programme, demonstrating help-seeking; this cohort cannot be considered representative of all carers, however, this is not the intention of qualitative research. This cohort could be considered a purposefully sampled collection of narratives of a phenomenon rarely studied yet essential for the wellbeing of people with a lived experience of EDs and their care communities. However, future research would benefit from a recruitment approach that would capture a broader array of carers and to expand this work for the benefit of contributing more stories to our understanding.

## Conclusions

This study supplements and extends a growing body of research calling for a transformation in working with people with enduring EDs, both for the experiencing individual and their carer. Carers’ narratives offer a starting point for reconsidering the traditional treatment methods by highlighting how carers moved beyond dominant models and forged ahead, made meaning of their experiences, and re-evaluated the caregiving role to respond to the uncertainty of their circumstances. Listening to lived experience requires bearing witness to the profound suffering and loss that coexists alongside acceptance and determination. In doing so, carers are reassured that their stories are heard, whilst acknowledging their critical importance to clinicians and researchers as they move towards new paradigms in the treatment of enduring eating disorders.

## What is already known on this subject?

Families and carers are often the primary source of support and treatment for people with eating disorders. This role can often lead to distress and burden. There is a paucity of information exploring the experiences of caring for someone with a severe and enduring eating disorder which may limit understanding and treatment approaches for this at-risk group.

## What this study adds?

This study sheds light on the importance of listening to the voices of carers, who are often silenced, yet have dedicated many years supporting their loved ones through the journey of eating disorders while experiencing feelings of guilt, personal failure, grief, despair, and a sense of disillusionment with current treatment approaches. Despite a myriad of negative experiences, carers also expressed feelings of hope, acceptance, commitment to their loved one, letting go and forging on. With severe and enduring anorexia nervosa having one of the highest mortality rates among other mental health conditions, there is an urgent need to listen and consider various individual experiences of people with this lived experience, and the people who care for them, and do more to transform the treatment paradigms ultimately leading to better support and outcomes.

### Supplementary Information

Below is the link to the electronic supplementary material.

Supplementary file1 (DOCX 27 KB)

## Data Availability

The interview data has limited availability as per ethics approvals, contact corresponding authors for more information.

## Abbreviations

AN: Anorexia nervosa
ARFID: Avoidant restrictive food intake disorder
BN: Bulimia nervosa
ED: Eating disorder
FBT: Family-based treatment
GP: General practitioner
SE-AN: Severe and enduring anorexia nervosa
