# Supplementary material for: “I don't know how, if, it's ever going to end”: narratives of caring for someone with an enduring eating disorder
Source: Eat Weight Disord. 2024 Jul 30;29(1):50. doi: 10.1007/s40519-024-01681-5 (PMC11289154; doi:10.1007/s40519-024-01681-5)
Supplement: Supplementary file 1 — Supplementary file1 (DOCX 27 KB) [file 40519_2024_1681_MOESM1_ESM.docx]

***“I don't know how, if, it's ever going to end”:* Narratives of caring for someone with an enduring eating disorder**

**Supplementary Material 1 – Interview Schedule**

Following an explanation of the research and some brief engagement, interviews began with the question: *Can you tell me about your story of caregiving, starting from the very beginning, as you define it, and taking me through to the present day?*

Prompts and questions were used throughout in the following ways:

- To elicit and clarify a timeline of events, e.g. *Could you tell me about what happened next?*
- To elicit further details, e.g. *Could you tell me more about that?*
- To understand associated emotions and the meanings carers ascribed to experiences, e.g. *What was that like for you?*

On occasion, the interviewer used reflective listening and paraphrasing techniques to convey to carers that their experiences had been heard, though contributions from the interviewer were minimal.

Once carers had described a narrative of experiences up until the present day, they were then asked about how they understood their role into the future (e.g., *What do you envision moving forward?).*

Finally, interviews were closed by asking participants: *What was it like for you to tell this story?*
